# Discovery of Antivirals Using Phage Display

**DOI:** 10.3390/v13061120

**Published:** 2021-06-10

**Authors:** Esen Sokullu, Marie-Soleil Gauthier, Benoit Coulombe

**Affiliations:** 1 Department of Translational Proteomics, Institut de Recherches Cliniques de Montréal, Montréal, QC H2W 1R7, Canada; Marie-Soleil.Gauthier@ircm.qc.ca; 2 Department of Biochemistry and Molecular Medicine, Université de Montréal, Montréal, QC H3C 3J7, Canada

**Keywords:** phage display, bacteriophage, antiviral, antigen-binding fragment, single-chain variable fragment, nanobody, intrabody, transbody, peptide

## Abstract

The latest coronavirus disease outbreak, COVID-19, has brought attention to viral infections which have posed serious health threats to humankind throughout history. The rapid global spread of COVID-19 is attributed to the increased human mobility of today’s world, yet the threat of viral infections to global public health is expected to increase continuously in part due to increasing human–animal interface. Development of antiviral agents is crucial to combat both existing and novel viral infections. Recently, there is a growing interest in peptide/protein-based drug molecules. Antibodies are becoming especially predominant in the drug market. Indeed, in a remarkably short period, four antibody therapeutics were authorized for emergency use in COVID-19 treatment in the US, Russia, and India as of November 2020. Phage display has been one of the most widely used screening methods for peptide/antibody drug discovery. Several phage display-derived biologics are already in the market, and the expiration of intellectual property rights of phage-display antibody discovery platforms suggests an increment in antibody drugs in the near future. This review summarizes the most common phage display libraries used in antiviral discovery, highlights the approaches employed to enhance the antiviral potency of selected peptides/antibody fragments, and finally provides a discussion about the present status of the developed antivirals in clinic.

## 1. Introduction

Since ancient times, infectious diseases have been one of the leading causes of death and disability worldwide. Besides the Plague of Athens and smallpox, which were disastrous on Greek and Aztec civilizations, humankind has faced several infections during history with deleterious impacts on health but also on economics and politics [1,2]. Among infectious diseases, those of viral origin predominate and pose a serious threat to global public health whilst spreading rapidly and internationally as a result of increased human mobility [2,3]. In addition to the re-emergence of known infections, such as the influenza A pandemics in 1918, 1957, and 1968, the emerging viruses which infect new host species place a substantial burden on global health [4]. Over the last four decades, newly emerging viruses have been continuously discovered, where more than 70% of them entered either directly from wildlife reservoirs or indirectly through domestic animals [5]. In this manner, the number of cases and mortality rates are critical outcomes reflecting the level of the burden. For instance, the first Ebola outbreak in 1976 was caused by a virus which originated from the rainforest in Africa and was transmitted to humans from bats [6]. The second and the most severe Ebola epidemic was reported in 2014, resulting in 11,323 deaths and 28,646 infected people in West African countries [7]. While the Democratic Republic of the Congo (DRC)’s 2018–2020 outbreak was the second largest Ebola outbreak globally, with 3481 confirmed cases and a 66% mortality rate, nowadays there is another ongoing outbreak in Guinea and DRC [8]. The human immunodeficiency virus (HIV), another virus originating from wildlife, was first identified in 1983 as the causative agent of the Acquired Immunodeficiency Syndrome (AIDS) and occurred as a result of transmission of a simian lentivirus from chimpanzee reservoirs to humans [9,10,11]. As of 2019, the World Health Organization (WHO) declared that HIV had infected 76 million people and caused the death of about 33 million people globally [12]. Coronaviruses are a large family of viruses that have caused severe outbreaks worldwide after spreading to humans from bats. Severe acute respiratory syndrome (SARS) coronavirus (SARS-CoV) was the first deadly coronavirus strain that emerged in November 2002 in China and spread rapidly to other countries, including Hong Kong, Singapore, Canada, the United States, and some European countries [13]. The SARS epidemic ended in 2003 and caused more than 8000 confirmed cases and approximately 800 deaths [14]. Nevertheless, in September 2012, a novel coronavirus strain, Middle East respiratory syndrome (MERS) coronavirus (MERS-CoV), emerged in Saudi Arabia. Two years later, an outbreak was reported in a total of 27 countries including Saudi Arabia, Egypt, France, the United Kingdom, and the United States [15,16]. Since 2012, MERS-CoV resulted in 2494 confirmed cases and 858 deaths with a mortality rate of 34.4%, higher than that of SARS-CoV (~10%) [15,16]. The latest outbreak of coronavirus emerged at the end of 2019 in Wuhan, China from a causative agent closely related to SARS-CoV, namely SARS-CoV-2, while the disease it causes was named COVID-19 (coronavirus disease) [17]. The COVID-19 outbreak spread internationally rapidly and was declared a pandemic by the WHO in March 2020 due to the alarming levels of spread, severity, and governments’ inaction [18,19]. At the time of writing this review, there have been 171,292,827 confirmed cases of COVID-19 including 3,687,589 deaths worldwide [20]. While the list can be extended to other viruses emerging from wildlife (e.g., Hendra virus (HeV), Nipah virus (NiV), and Marburg virus), the threat to public health continuously increases and makes the development of effective treatments crucial to combat emerging viral infections [21,22,23].

Vaccination is the cornerstone in protecting individuals from viral diseases and preventing the emerging viral infections [24]. However, there are circumstances in which vaccination is not sufficient to reduce the infection burden, leading to the need of antiviral drugs. Low vaccine coverage in the society, lack of protective immunogenicity among older or immunocompromised people, and mismatch between vaccine and circulating strains due to virus mutations would be some examples of such possible circumstances [25,26]. Moreover, the lack of vaccines for some viruses, such as HIV and hepatitis C virus (HCV), also necessitates the development of effective antiviral drugs [27]. Since the approval of the first antiviral drug “idoxuridine” by the FDA in 1963, treatment of the infected patients with antivirals has been adjunct to vaccination, if not the only available treatment for some viruses (e.g., HIV, HCV, and herpes simplex virus (HSV)) [28,29]. There are various organic compounds used as drugs, including small organic molecules and biologics [30]. In their extensive review on FDA-approved antiviral drugs, Chaudhuri et al. reported that virus-targeting small-molecule antivirals (e.g., peramivir, simeprevir sodium, and docosanol) represent a large majority of the approved antiviral drugs, while the number of large-molecule biological candidates (e.g., interferons, oligonucleotides, monoclonal antibodies, and peptides) has recently increased [31]. Among these biologics, antibodies and peptides have gained particular attention in the modern drug market because of their high specificity which is mostly lacking in small organic molecules [30,32]. Protein-based drug molecules have benefited from the advancements of the techniques used in drug discovery pipelines. After the introduction of target-based drug discovery approaches, time consuming trial-and-error processes were replaced with high throughput library screenings to identify highly specific drug molecules [33]. Due to its simplicity, cost effectiveness, and speed, phage display has been one of the most widely used screening methods and has led to small peptide and antibody drugs discovery [34]. The technology involves the fusion of peptides/proteins into the genome of bacteriophages (phages) to be expressed on the phage surface as fusions to the coat proteins [35,36]. The physical link between the displayed peptides/proteins and their encoded DNA sequence enables identification of high-affinity binders through DNA sequencing after the phage display combinatorial library screening against the target of interest [37,38,39]. After the first description of phage display peptide libraries in 1985 by George Smith [40], the technique was used for the construction of antibody libraries by Gregory Winter and his colleagues, who pioneered the development of antibody drugs by phage display technology [41,42]. Phage display technology as well as the different phage display platforms used in drug discovery have been reviewed elsewhere and will not be further discussed herein [43,44,45]. In this review, we summarize the most common protein scaffolds used in construction of phage display libraries and their current applications in antiviral discovery. In addition, we discuss the present status of various developed antivirals in clinical research and the challenges they face in penetrating the clinic.

## 2. Phage Display Libraries Used for Antiviral Discovery

### 2.1. Peptide Libraries

Peptides are attractive drug candidates because of their distinct biochemical and therapeutic characteristics [46]. Peptides offer some advantages similar to those of small organic molecules, such as good tissue and organ penetration, but exhibit higher target specificity than small molecules [47]. However, their lower production cost, lower immunogenicity, higher activity per mass, and greater storage stability are all considered advantages over larger biologics such as antibodies [47,48].

The most common phage display peptide libraries are based on the filamentous phages, so-called fd, f1, and M13, in which the peptide sequences are fused to either the minor coat protein (pIII), or major coat protein (pVIII) on the phage surface [49]. Although it is common to screen in-house phage display libraries, the commercial peptide libraries (e.g., New England BioLabs (NEB) and MoBiTec GmbH) have also been used to develop peptide-based antivirals [50,51,52]. For instance, 7-mer and 12-mer linear peptide libraries of NEB have been widely used to identify antivirals for various viruses (e.g., avian infectious bronchitis virus (IBV), dengue virus serotype 2 (DENV-2), Macrobrachium rosenbergii nodavirus (MrNv), Japanese encephalitis virus (JEV), porcine reproductive and respiratory syndrome virus (PRRSV), porcine epidemic diarrhea virus (PEDV), transmissible gastroenteritis (TGE) virus, infectious salmon anemia virus (ISAV), bovine ephemeral fever virus (BEFV), influenza B virus, and Mink enteritis virus (MEV)) (Table 1) [53,54,55,56,57,58,59,60,61,62,63,64]. Cao et al. [65] employed a 12-mer phage display peptide library to develop antivirals against PEDV, the causative agent of persistent diarrhea in swine. Library screening against the MAb-5E12 antibody which can bind to the spike (S) protein of PEDV resulted in three different peptide sequences (LMQINPTYYQLM, WSFNPSTYTIAG, and HDFVADMYQLAQ) with specific binding affinity for MAb-5E12. Alignment of the selected peptides with the native S1 protein revealed high sequence similarity to a putative linear, antigenic epitope of the PEDV S1 protein (^201^MQYVYTPTYYML^212^). Two peptide sequences (LMQINPTYYQLM and WSFNPSTYTIAG) with a consensus motif “PxxY” interacted with cell-surface receptors and blocked the adsorption of virus to the cells treated with peptides prior to virus addition. Although MAb 5E12 had no neutralizing activity against PEDV, two MAb-5E12-recognizing peptides (LMQINPTYYQLM and WSFNPSTYTIAG) significantly reduced the virus production in vitro by inhibiting the adsorption of PEDV to cell-surface receptors. The size of peptides, which is smaller than that of antibodies, was suggested to allow better binding to the cellular receptors and induce inhibitory activity. In another report, Lü et al. screened a 7-mer phage display peptide library against HCV envelope protein E2 from genotype 1a [66]. Cell entry inhibition assays were performed with the selected peptide (WPWHNHR) by employing HCV particles bearing E proteins of genotype 1b and genotype 2a. The peptide WPWHNHR inhibited the entry of HCV particles from both genotypes into Huh7.5 cells, suggesting potential applications of the peptide as HCV antivirals, regardless of the genotype of the virus. Type I interferon (IFN), a therapeutic agent to treat viral infections, was also used as a target to screen 7-mer phage display libraries to discover antiviral peptides [67]. Library screening was performed against IFNα-2b-expressing WISH cells in the presence of vesicular stomatitis virus (VSV). While VSV infection killed the majority of WISH cells, the surviving cells were expected to have phage clones with antiviral activity. In fact, competitive elution of phage clones by IFNa-2b resulted in two phage clones displaying peptides with sequence similarity to IFNα-2b and mimicking the antiviral activity of IFNα-2b cooperatively.

Cyclic peptide libraries from NEB are another format of phage display peptide libraries widely used in antiviral drug development [68,84,85,86]. Cyclic peptides have several features making them attractive alternatives to linear peptides. Firstly, the constrained structures of cyclic peptides reduce their flexibility and result in lower entropic penalty upon binding to their targets [87]. Reduced flexibility usually results in high selectivity and specificity, as well [88]. Furthermore, in comparison to linear peptides, they are relatively stable and less prone to proteolytic degradation, which makes them more favorable as therapeutic agents [89]. A cyclic phage display peptide library was used by Hall et al. in order to develop antivirals against Andes virus (ANDV), which belongs to the genus Hantavirus [90]. Library screening was performed against purified UV-treated ANDV strain CHI-7913. In order to achieve high specificity, phage clones were eluted by monoclonal antibodies specific to recombinant ANDV Gn glycoprotein (antibody 6B9/F5) or Gc glycoprotein (antibody 6C5/D12). The three most potent synthetic cyclic peptides (CPSNVNNIC, CPMSQNPTC, and CPKLHPGGC) inhibited ANDV entry in a dose-dependent manner, whereas the 50% inhibitory concentration for each of the peptides (IC_50_) was in the range of 10 μM. The specificity of the peptides for ANDV was also demonstrated in inhibition assays employing other hantaviruses (Sin Nombre hantavirus (SNV), Hantaan virus (HTNV), and Prospect Hill virus (PHV)). In another report, the NS5B protein of hepatitis C virus (HCV), which possesses RNA-dependent RNA polymerase activity, was employed as a target molecule to develop antiviral peptides for HCV [69]. Screening of a cyclic 7-mer peptide library resulted in three cyclic peptides (CFPWGNTWC, CFPWGKEYC, and CFPWGNQWC) with inhibitory activity on RNA-dependent RNA polymerase. Polymerase assays indicated that these peptides blocked the formation of the pre-elongation complex, which consists of HCV polymerase bound to RNA substrate, or a transition step required for the pre-elongation complex to catalyze RNA synthesis. The peptides did not affect RNA synthesis once the pre-elongation complex was formed. 

In addition to combinatorial peptide libraries, phage display libraries constructed from cDNAs have been also employed in the discovery of antiviral peptides. For instance, a murine brain cDNA phage display library was screened against the West Nile virus (WNV) envelope (E) protein to select a peptide with inhibitory activity on virus–host cell interaction [70]. The neurovirulent nature of WNV suggested that brain tissue cDNAs might encode a receptor/ligand with high affinity for the WNV E protein. The selected P9 peptide CDVIALLACHLNT could specifically bind to WNV and inhibit virus infectivity in vitro with an IC_50_ value of 2.6 μM. The P9 peptide also protected mice from lethal infection of WNV. 

Many efforts have been made to develop antiviral peptides against hepatitis B virus (HBV), one of the most widely studied viral agents. The initial step of HBV infection is the attachment of virions to specific cell-surface receptors via the viral envelope proteins [91]. The virus-derived large surface protein of the HBV virion contains an N-terminal PreS domain which is responsible for viral attachment and has thus been considered a potential target for antiviral drug discovery [92]. A 12-mer phage display peptide library was screened against recombinant PreS protein and resulted in peptide sequences exhibiting a short consensus sequence (W/FTXWW/F) with PreS- and HBV-binding activities [93]. It was shown that preincubation of HBV-positive serum samples with the consensus peptide reduced the binding of HBV to LPL-expressing THP-1 cells. Wang et al. also identified a group of HBV PreS1-binding peptides by screening a 12-mer phage display peptide library [71]. Among them, the P7 peptide (KHMHWHPPALNT) was able to inhibit the interaction of HBV PreS1-positive sera with the antibody of PreS1 in ELISA assay format. P7 peptide was suggested to cover key functional sites of PreS1 protein, interacting with its antibody. In another report, A5 peptide (LKKKW) was selected by screening a phage display library against maltose-binding protein fusion of PreS_11–65_ [94]. Optimization of the PreS1-binding affinity of A5 peptide by a comprehensive mutagenetic analysis resulted in B10 peptide (LRNIR) showing a ~1.5-fold higher affinity than that of A5. Moreover, an immunofluorescence study demonstrated that both peptides could significantly reduce the attachment of HBV to HepG2 cells. The interaction of HBV core antigen (HBcAg) with HBV surface antigens is crucial for virus assembly; therefore, it has been another target for antiviral development [95]. For instance, phage display library screening against recombinant HBcAg (aa 3–148) resulted in LLGRMK peptide with specific binding affinity for full-length HBcAg [96]. The addition of two flanking amino acids of the pIII coat protein improved the binding affinity of peptide ALLGRMKG that inhibited the interaction between L surface antigen and HBcAg with the half maximal inhibitory concentration (IC_50_) of 10 μM.

The lack of vaccine for HIV makes this virus an important target for antiviral drugs with reduced adverse effects [97]. Several viral components critical for HIV infection, such as HIV-1 Gag polyprotein and HIV integrase (IN), have been targeted for drug development [72,98]. The gp120 glycoprotein, a part of the viral envelope, has been one of these targets [99]. Gp120 binds to CD4 cell-surface receptors and facilitates HIV entry into host cells [100]. Therefore, it is suggested that peptides with gp120 binding affinity could inhibit HIV infection by blocking the interaction of virus with cell receptors. Chevigne et al. constructed a phage display peptide library derived from the plasma of long-term survivors that demonstrated strong IgG responses against gp120 [101]. Library screening against recombinant X4 virus gp120 protein resulted in an LRTV-1 peptide (VYFCAKVPQVDFWRRIFDYWCRG) that showed sequence homology with the chemokine receptors CXCR4 (11/23) and CCR5 (7/23). Inhibition assays demonstrated that the LRTV-1 peptide inhibited the infection of X4 (III_B_) and R5 (BaL) viruses in MT-4 and MT4-CCR5 cells with IC_50_ values of 45 ± 1.6 and 58 ± 1.8 μmol/L, respectively. Chemokine receptors (e.g., CCR5 and CXCR4) are used as coreceptors for HIV entry into host cells along with CD4 receptors [102]. Consequently, the interaction of HIV with chemokine receptors has been an interesting target for antiviral drug development [103]. Towards that goal, Hartley et al. constructed a phage display library by using the cDNA encoding human RANTES, a natural chemokine ligand with inhibitory activity on HIV entry [104]. N-terminally mutated RANTES variants were created by partial and complete randomization in certain positions. Library screening against adherent live Chinese hamster ovary (CHO-CCR5) and human embryonic kidney (HEK-CCR5) cells led to P1 (LSPVSSQSSA) and P2 (FSPLSSQSSA) peptides that were more potent than RANTES. The most active P2 peptide exhibited an IC_50_ value of 600 pM in a viral coat-mediated cell fusion assay as well as a complete cell protection against HIV-1 strains at 10 nM concentration. Integrase, a crucial enzyme catalyzing the integration of viral DNA into cellular DNA, has been another target to develop HIV antivirals [105]. Phage display peptide libraries were screened against the C-terminal fragment of lens epithelium-derived growth factor, LEDGF/p75, which is an interactor of the HIV integrase [89]. In order to study the antiviral activity, the selected cyclic peptides, CP64 (CVSGHPLWC) and CP65 (CILGHSDWC), were expressed as monomeric red fluorescent protein fusions in HelaP4 cells. The infection of cells with HIV-1 molecular clone NL4.3 did not decrease viral production; however, CP65 reduced the infectivity of the newly produced progeny virions. The development of drug resistance against anti-HIV drugs targeting virus-encoded proteins is a common phenomenon, therefore resistance selection against CP65 was also investigated. Interestingly, serial passaging of HIV-1NL4.3 in HeLaP4 cells expressing CP65-mRFP did not yield resistant strains. 

### 2.2. Antigen-Binding Fragment (Fab) Libraries

**Figure 1:**
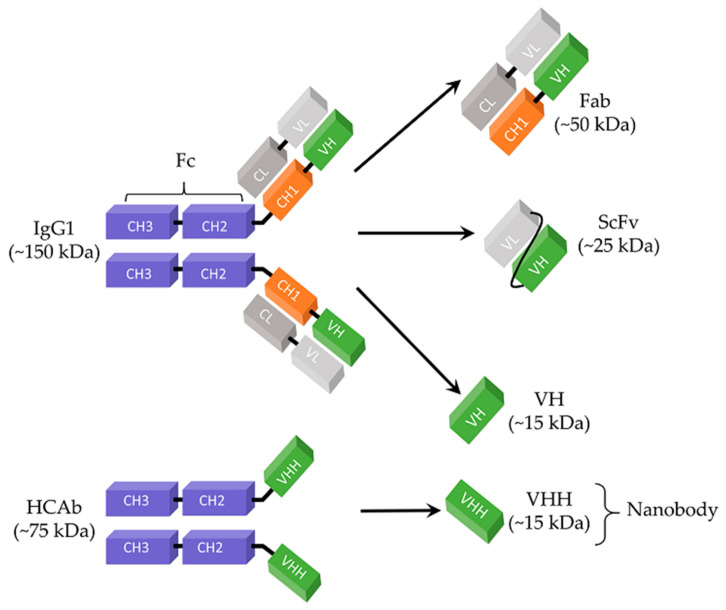
Schematic representation of antibodies and antibody fragments. IgG1, immunoglobulin G1; HCAb, heavy chain-only antibody; Fc, crystallizable fragment; Fab, antigen-binding fragment; ScFv, single-chain variable fragment; CH1–CH2–CH3, constant domains of heavy chain; CL, constant domain of light chain; VH, variable domain of heavy chain; VL, variable domain of light chain; VHH, variable heavy chain of HCAb.

Since the discovery of hybridoma technology in 1975 [106], monoclonal antibodies (mAbs) have been important therapeutic agents because of their features, such as unique target specificity, ability to target a wide range of molecules, and long serum half-life [43,107,108]. The scientific and ethical concerns presented by antibodies derived from animal immunization made researchers investigate alternative technologies [109]. Production of antibody libraries by advanced recombinant technologies has been an attractive approach to obtain access to an enormous repertoire of structurally diverse antibodies [109]. In this regard, phage display technology has been a powerful tool and widely used in antibody library construction since its first demonstration in 1990 by McCafferty et al. [110,111]. Antigen-binding fragment (Fab), the first therapeutic antibody that entered clinical development, has been one of the formats used in phage display antibody library construction [112]. Fab consists of an antibody light chain (VL + CL domains) and an antibody heavy chain (VH and CH1 domains) linked by a disulfide bond (Figure 1) [113]. Their relatively small size (~50 kDa) enhances the tissue penetration of Fabs as compared to mAbs (~150 kDa), while the risk of immunogenicity and their half-life are reduced by lack of an Fc domain [113]. Because of their monovalent format, Fabs can be easily produced in their monomeric form and this feature enables their use for different purposes, such as characterization assays [114,115].

Phage display Fab libraries can be synthetic or natural repertoires which are constructed from B cells of immunized or non-immunized donors. Construction of combinatorial libraries from immunized donors allows the user to have access to a monoclonal antibody specific for that particular immunogen [116,117]. In fact, Zhang et al. constructed a phage display Fab library from the bone marrow of long-term nonprogressor HIV-infected individuals to identify cross-reactive neutralizing antibodies for HIV infection [118]. For this purpose, they employed sequential antigen panning (SAP) in which complexes of two different recombinant envelope glycoproteins from HIV isolates 89.6 and IIIB (gp140_89.6_ and gp140_IIIB_) with CD4 receptor and the envelope glycoproteins alone were used as antigens for different rounds of library screening. The resulting antibody, m18, bound to gp120 from the primary isolates 89.6 and JR-FL with a *Kd* of 1 nM, and to the TCLA strain IIIB with 0.1 nM in ELISA. In a pseudotype virus assay, the cell entry of most HIV isolates was inhibited by m18 at concentrations up to 100 μg/mL, while the 50% neutralization was achieved at concentrations ≤0.1 mg/mL for 11 of the 15 primary isolates tested. Neutralizing activity of the m18 Fab fragment was comparable to the potent broadly HIV-neutralizing human monoclonal antibody Fab X5, which neutralized 13 out of 15 primary isolates with similar activity (IC_50_, ≤0.1 mg/mL). In another report, a Fab library was constructed from the bone marrow of a chimpanzee previously infected with all five recognized hepatitis viruses (A to E) [74]. The library screening against recombinant hepatitis E virus (HEV) ORF2 protein from Pakistani strain SAR-55 (SAR-55 ORF2) led to HEV#4 and HEV#31 antibodies having specific binding affinities for the SAR-55 ORF2 with *Kd* values of 1.7 and 4.5 nM, respectively. Fabs and the SAR-55 strain of HEV were incubated before their inoculation, and the neutralization activity of Fabs was determined in rhesus monkeys (Macaca mulatta). No HEV infection occurred in the monkeys that received HEV incubated with either HEV#4 or HEV#31, whereas four monkeys developed HEV infection in the control group which received HEV incubated with either irrelevant HBV#8 Fab or chimpanzee 5835 preimmune plasma. 

### 2.3. Single-Chain Variable Fragments (ScFv)

ScFv is another recombinant antibody format which has been used in antiviral development. It consists of the smallest high-affinity antigen-binding site of an antibody, the variable heavy (VH) chain and variable light (VL) chain, connected by a flexible polypeptide linker (e.g., (Gly4Ser)_3_) (Figure 1) [119]. Preservation of the binding activity of the parental antibody, increased penetration capacity in dense tissues, and convenient expression in a broad range of hosts (e.g., bacteria, yeast, plants, and mammalian cells) each make ScFv an attractive antibody construct for therapeutic agent development [120,121]. Yet, rapid blood clearance and poor retention in the target tissue are limitations associated with the small size of ScFvs (~25 kDa) [122].

Various synthetic and natural ScFv phage display antibody libraries have been constructed and employed in the discovery of antivirals [123,124,125,126]. For instance, a synthetic human ScFv phage display library was screened against Ebola filovirus (EBOV)’s viral protein 35 (VP35), which is a cofactor of the viral RNA polymerase complex as well as a double-stranded RNA-binding protein interfering with the host immune response by blocking the interferon (IFN)-mediated antiviral activity [127]. To evaluate the inhibition activity of selected ScFvs, A549 cells expressing IFN-β and EBOV VP35 protein were co-transfected with the anti-VP35 scFv expression plasmid. Two of the five ScFvs tested, F9 and E1, interfered with the VP35-dependent inhibition of IFN activity by binding to VP35 proteins. They destroyed the inhibition of the IFN-β production generated by dsRNA, after VP35 expression. A similar study was also performed by Amatya et al. in which a synthetic Fab phage library was screened against the recombinant mVP35 IFN inhibitory domain (IID) protein of Marburg virus [75]. Although a synthetic Fab library was employed, the selected Fabs were expressed in ScFv format to investigate their inhibitory activity. Coexpression of the ScFv H3 fragment with the components of the viral polymerase complex inhibited the Marburg polymerase complex in a dose-dependent manner. Two alternative inhibition mechanisms were suggested: (i) generation of a steric hindrance by ScFv H3 at the interface of mVP35 IID required for viral RNA synthesis or (ii) unfavorable conformational change in mVP35 IID structure upon binding to ScFv H3. In order to develop neutralizing antibodies against the H5N1 influenza A virus, Maneewatch et al. employed a naïve human ScFv phage display library [128]. The library was screened against recombinant haemagglutinin (HA), which plays critical role in receptor binding and membrane fusion of influenza virus. The selected anti-HA ScFv could neutralize the infectivity of all tested strains of the H5N1 viruses in the Madin–Darby canine kidney (MDCK) cell assay. Moreover, four intraperitoneal injections of 2 mg/kg/dose ScFv rescued C57BL/6 mice infected with heterologous H5N1 virus. While the virus recovery from the internal organs was significantly reduced by administration of ScFv at 5 and 10 mg/kg, a reduction in the lung histopathology of the infected mice was also observed. In another study, Zhao et al. constructed an ScFv phage display library from the peripheral blood lymphocytes (PBL) of donors immunized with rabies virus vaccine [129]. The library screening was performed against the linear neutralizing epitope of rabies virus Gp protein (PPDQLVNLHAFVRSDEIEHLVVEE) and resulted in T166 and F21 ScFvs with neutralizing activities for rabies virus. Plaque reduction neutralization assay demonstrated that T166 and F21 ScFvs could inhibit plaque formation of three different rabies virus strains (CVS-II, CQ92, and SBD) in Vero cells.

### 2.4. Nanobodies

Nanobody is the term used to refer to the antigen-binding fragments derived from heavy chain-only antibodies (HCAb) found in the sera of Camelidae (e.g., camels, llama, and vicugna) [130]. Unlike conventional monoclonal human antibodies (mAbs), HCAbs are devoid of both the entire light chain (CL + VL) and the first constant domain of the heavy chain (CH1), making their size (75 kDa) roughly half of a conventional mAb (150 kDa) (Figure 1) [131,132]. As a consequence of this unique structure, the interaction of HCAb with its antigen occurs through the only variable domain of the heavy chain, the so-called VHH (Figure 1) [133]. VHHs can be produced as stable and soluble recombinant antibody fragments and used as therapeutic agents [134]. Because of their single-domain nature, VHHs are considered as the smallest antigen-binding fragments. In fact, VHHs are designated as “nanobodies” because of their nano-scale dimension (2.5 nm in diameter and 4.2 nm in length) [135]. Nanobodies have been promising alternatives to conventional mAbs which are suffering from limited tissue penetration due to their large size and the host immune response [136]. While their small size enhances the tissue penetration and enables them to target epitopes inaccessible to traditional mAbs, lack of an Fc domain reduces the half-life and risk of immunogenicity of nanobodies [136,137]. Although Fabs and ScFvs also have the same advantage of small size in clinical applications, nanobodies are considered better candidates because of their robust and stable structure, high solubility, and high production yield in microbial expression systems [138]. 

Development of nanobodies as antiviral therapeutic agents has been the objective of several studies. Phage display VHH nanobody libraries have been generated from the blood cells of immunized and non-immunized animals of the Camelidae family, such as the alpaca, dromedary, and camel [139,140,141]. Several viral components, ranging from whole viral particles to nonstructural proteins, have been employed as targets in nanobody library screening. For instance, Tarr et al. constructed a phage display VHH library derived from an alpaca immunized with the HCV E2 glycoprotein [142]. Library screening against E2 protein resulted in D03 nanobody that recognized a novel epitope localized in the cell receptor (CD81)-binding region of E2 and overlapped with the epitopes of several broadly neutralizing anti-HCV E2 mABs. D03 could neutralize the majority of the tested primary HCV isolates with IC_50_ values ranging from 1 to 10 μg/mL. Moreover, the D03 nanobody was able to inhibit cell-to-cell transmission of HCV, which is resistant to some human neutralizing antibodies. The smaller size of D03 (15 kDa) as compared to other antibody/fragments, and its specific binding mode to E2 protein, were suggested as a possible explanation for its outperformance. On the other hand, the opportunity of using nanobodies as intrabodies has made these fragments more interesting for the development of antivirals targeting nonstructural viral proteins. Intrabodies are antibody fragments designed to be expressed intracellularly and to alter the functions of cellular antigens by modifying pathways or redirecting the target molecule to a new cellular compartment [143]. In that respect, nonstructural viral proteins with RNA-dependent RNA polymerase (RdRp) activity and 3C-like serine protease (3CLSP) activity have been used as targets for the development of antiviral intrabodies based on VHHs [144,145]. For instance, nonstructural NS5B protein of Bovine viral diarrhea virus (BVDV) with RdRp activity was employed as a target for the screening of nanobody libraries to discover antiviral drugs against BVDV [146]. A phage display VHH library was constructed from blood cells of Bactrian camel immunized with soluble recombinant NS5B protein. In order to study the antiviral activity of the selected nanobodies, Madin Darby Bovine Kidney (MDBK) cell lines stably expressing the nanobodies were established using lentiviral packaging technology. Virus infection experiments showed that the intracellularly-expressed novel nanobody, so-called Nb1, could suppress BVDV infection in MDBK cells without any obvious cytopathic effects at 48 h post infection. While Western blotting confirmed the abrogation of NS5B protein expression by Nb1 nanobody, prohibition of BVDV replication was shown in titration assays. In fact, Nb1 nanobody expression in MDBK cells resulted in a ~10,000-fold and ~1000-fold decrease in viral titers at MOIs of 0.1 and 1, respectively. Moreover, the authors also showed that Nb1 intrabodies could promote cell survival at MOIs of 0.01, 0.1, and 1, suggesting antiviral activity of Nb1 intrabodies against BVDV. 

Despite their several advantages, the camelid origin of nanobodies presents a risk of immunogenicity limiting their use in humans [147]. Therefore, human single-domain antibodies consisting of one variable domain of antibody heavy chain (VH) have been considered as an alternative to nanobodies because of their similar small size (~15 kDa) [148]. However, human VH domains are naturally paired with VL domains and their production in the absence of VL results in poor biophysical properties, as well as low solubility [149]. Several research groups have designed new scaffolds based on camelid VHH and human VH domains in order to generate phage display single-domain antibody libraries, resulting in human VHs with enhanced stability and solubility [150,151]. In this regard, Wu et al. constructed a phage display human single-domain antibody library in order to discover SARS-CoV-2-specific antibodies as therapeutics [81]. The M36 antibody fragment, a highly soluble and stable VH with HIV-1-neutralizing activity, was used as a reference scaffold for library construction. The IMGT database was searched for human immunoglobulin heavy-chain variable region (IGHV) alleles that had the same germline framework regions (FR1, FR2, or FR3) as m36. After expressing several IGHV alleles in bacterial cells, germline 3 − 66 × 01 was found to have the best biophysical properties. Its framework regions were used as the scaffold, and heavy-chain complementarity-determining regions CDR1, CDR2, and CDR3 of several naïve antibody libraries were grafted onto it. Library screenings were performed against S1 subunit and receptor-binding domain (RBD) of SARS-CoV-2 S glycoprotein and resulted in five different antibody groups recognizing distinct epitopes on RBD. The antibodies n3130 and n3088 recognizing a “cryptic” epitope located in the spike trimeric interface neutralized live SARS-CoV-2 with an IC_50_ value of 4.0 and 2.6 mg/mL, respectively, without any cytopathic effect. Compared to non-neutralizing mAb CR3022, which can recognize the same epitope on SARS-CoV-2, VH antibody fragments could neutralize the virus infection and their inhibitory activity was attributed to their small size.

### 2.5. Other Protein Scaffolds

Compared to small linear peptides, which lack pre-existing conformations or cyclic peptides, mimicking only a single β-turn, proteins are more prone to adopt well-defined structures leading to high affinity and specificity [152]. Therefore, in addition to antibody fragments, various proteins have been used as scaffold to construct phage display libraries. Minibodies are small β-protein scaffolds derived from the variable heavy-chain domain of the mouse antibody McPC603 [153]. Along with the variable domain, they consist of exposed hypervariable regions H1 and H2, which can be randomized to construct phage display libraries [154]. The β-sheet framework of a minibody provides a stable scaffold for library construction and facilitates the proper folding of the selected ligand [155]. Display of pancreatic secretory trypsin inhibitor (PSTI) variant proteins on M13 phage pIII coat protein offers another stable protein scaffold for phage display library construction. PSTI is a 56-amino-acid protein consisting of six cysteine residues involved in disulfide bridges [156]. Display of PSTI variants on the phage surface enables the design of inhibitors with new protease specificities [157]. Dimasi et al. employed both PSTI and minibody libraries to discover the inhibitors for NS3 protease, a structural protein essential to HCV replication [158]. The inhibitory activity of selected a minibody (MBip) was tested on an entire NS3 protein due to the absence of cell-based HCV replication systems. MBip demonstrated an inhibitory activity for the NS3 enzyme with an IC_50_ value of 1 μM. In the case of the PSTI library, screening resulted in 10 different variants and only one of them, hPSTI-C3, could be expressed in bacterial cells and refolded properly. Inhibitory activity of hPSTI-C3 on the NS3 protease was also observed (IC_50_, 1.2 mM). However, low expression levels of disulfide-rich PSTI proteins remains a limitation for PSTI-based protease inhibitors discovery. 

Designed ankyrin repeat proteins (DARPins) is another example of a protein scaffold employed in the construction of phage display libraries. Ankyrin repeat is a 33-amino-acid residue repeating motif found in more than 400 proteins in nature [159]. Ankyrin repeat proteins are built from tightly packed repeat domains usually consisting of 4 to 6 repeats with a large solvent-accessible surface [160]. High thermodynamic stability against unfolding, high solubility, and high expression levels of soluble form in bacterial cells are all distinct advantages of DARPins over many other protein scaffolds [160]. For instance, a phage display ankyrin library was used to discover antivirals for HIV-1 through a screening against a fusion protein consisting of matrix (MA) and capsid (CA) domains of the HIV-1 Gag precursor [161]. The selected Gag-specific artificial ankyrin, AnkGAG1D4 (*K_d_*, ~1 μM), contained three ankyrin repeats flanked by an N-cap and C-cap. AnkGAG1D4-expressing SupT1 cells showed reduced permissiveness to HIV-1 infection. The antiviral effect was shown to occur at post-integration steps of the HIV-1 life cycle, involving the Gag protein assembly and budding machinery.

## 3. Strategies to Increase the Potency of Phage Display-Selected Antivirals

### 3.1. Polyvalent Presentation of Antiviral Peptides

Attachment of a virus to its host cells occurs via simultaneous binding of multiple viral surface coat proteins to multiple receptors on the host cell surface [162]. Because of this polyvalency, virus–host interactions can be collectively much stronger than the corresponding monovalent viral protein–cell receptor interaction [162]. As a consequence, the use of monomeric antiviral molecules may not efficiently block the polyvalent virus–host interactions [163]. Polyvalent inhibitor systems, which promote cooperative virus–inhibitor interactions, have been proposed to overcome the polyvalency problem and enhance inhibitory activity [163]. Polyvalent presentation of antiviral peptides on SiMAG/1-carboxyl magnetic silica particles (100 μm) has been one of these approaches applied to improve the inhibitory activity of antivirals [164]. The cyclic peptides selected by phage display library screening against inactivated SNV were chemically conjugated to silica nanoparticles at different nanoparticle-to-virus ratios (4:1 and 20:1). Two peptides, CLVRNLAWC and CQATTARNC, showed enhanced inhibition over the free format when presented on nanoparticles at a 4:1 nanoparticle-to-virus ratio (9.0 to 32.5 and 27.6 to 37.6%, respectively), whereas CQATTARNC-mediated inhibition reached more than 50% when nanoparticles were used at a 20:1 ratio relative to virus. It is important to note that the authors used monovalent peptides at a concentration of 1 mM in inhibition assays. With respect to the polyvalent presentation, the peptide concentration on a molar nanoparticle basis and on peptide molar equivalents were calculated at approximately 46 pM and 61.6 fM, respectively, showing the impact of polyvalent presentation in inhibitory activity of peptide inhibitors. 

Antiviral peptides have also been integrated in molecular assemblies to improve their inhibitory activities [165]. Matsubara et al. employed this approach for 15-mer peptides exhibiting binding affinity for sialic acid (Neu5Ac)-containing glycoconjugates found on the surface of certain tracheal epithelial cells and recognized by the envelope glycoprotein, hemagglutinin (HA), of influenza virus to initiate viral infection [73]. Despite the specific binding of selected peptides c01 (GWWYKGRARPVSAVA) and c03 (RAVWRHSVATPSHSV) to Neu5Ac-containing structures (Neu5AcR2-3Galβ1-4Glcβ1-1′Cer (GM3); Kd values of 1.5 and 0.41 μM, respectively), they did not show any inhibitory activity against infection of MDCK cells by H1N1 and H3N2 viruses. To enhance peptides’ binding affinity, N-stearoyl amides (C18-peptide) were formed by modifying the peptides’ N-terminal with an alkyl group (C18). The molecular assembly of the N-stearoyl peptide amides resulted in peptide assemblies, C18-c01 and C18-c03, with strong inhibitory activity on infection of MDCK cells by H1N1 influenza virus, with IC_50_ values of 3.2 and 6.5 μM, respectively. 

Production of antiviral peptides as multiple copies in tandem has been another approach to improve their binding affinity and inhibitory activity. Zhou et al. initially employed maltose-binding protein (MBP) as a fusion tag to enhance the solubility of 12C peptide (LHWDFQSWVPLL), which exhibited binding affinity for orange-spotted grouper nervous necrosis virus (OGNNV) [166]. In order to improve the binding affinity of the peptide, MBP was fused with tandem repeats of 12C. In fact, MBP–T12C protein that was generated by fusing MBP to triple 12C peptide exhibited a 9-fold higher OGNNV-binding affinity than that of MBP fused with single 12C peptide. Moreover, the treatment of SB fibroblasts with the MBP–T12C protein at a 400 μg/mL concentration significantly reduced viral entry, but not viral propagation within the cells. The difficulties in intracellular delivery of MBP–T12C protein were suggested to create the inefficient inhibition of OGNNV propagation.

### 3.2. Modification of the Peptide Backbone to Enhance Stability

Short serum half-life has been the major drawback of peptide-based therapeutics, limiting their clinical applications [167]. Poor bioavailability of peptides arises from their rapid degradation by endogenous proteases and modifications are introduced to peptide backbones to enhance their proteolytic stability [168]. Amino acids, except glycine, are chiral molecules existing in either levorotatory (L) or dextrorotatory (D) forms in nature, yet the former is mostly used in construction of protein structures in life [169]. As a result of the changes in backbone side chain connectivity and geometry, D-proteins cannot be recognized by L-proteins such as proteases [170]. This feature makes D-proteins resistant to proteolytic degradation and suggests the introduction of D-amino acids to the peptide backbones as a promising approach to improve peptide stability in cells. Liu et al. investigated the effect of chirality on the antiviral activity of p9 peptide selected by screening a 12-mer phage display peptide library against recombinant RNA-dependent RNA polymerase (NSP9) protein of PRRSV [171]. The treatment of MARC-145 cells with the dextral p9 (D-p9) and levorotatory p9 (L-p9) peptides demonstrated that D-p9 had higher inhibition on an intracellular PRRSV copy number than that of L-p9 determined with IC_50_ values of 16.12 and 56.47 μM, respectively. 

Besides synthesis of D-peptides by direct translation from already known L-peptide sequences, antiviral D-peptides have also been identified by a modified phage display technique called mirror-image phage display [172,173]. In this method, the target of interest is chemically synthesized with D-amino acids to generate a mirror image of the naturally occurring L-enantiomeric target [174]. Afterwards, a phage library displaying L-peptides is screened against the D-target and the selected L-peptides are chemically synthesized with D-amino acids to obtain their mirror image [174]. By symmetry, the resulting D-enantiomeric peptides can specifically bind to the natural target in L-enantiomeric form [174]. Eckert et al. used mirror-image phage display to discover D-peptide, which can inhibit HIV-1 cell entry by targeting the hydrophobic pocket of the N-peptide coiled-coil region of envelope glycoprotein gp41 which promotes viral entry by mediating the fusion of viral and cellular membranes [173]. The target molecule (D-IQN17) was designed by fusing a soluble trimeric coiled coil to the portion of the N-peptide in the gp41 hydrophobic pocket and was chemically synthesized with D-amino acids. Screening of a phage display peptide library resulted in a consensus sequence (CXXXXXEWXWLC) with binding affinity to D-IQN17 protein. Chemical synthesis of selected peptides with D-amino acids led to D-peptide having binding affinity for the natural L-enantiomeric form of the target protein. The inhibitory activity of the D-peptides was tested in luciferase-based HIV-1 infection assay in which all peptides inhibited HIV-1 entry into HOS-CD4/Fusin cells with IC_50_ values at a range of 11 to 270 μM. Welch et al. took this work further and constructed a comprehensive library of CXXXXXEWXWLC sequences in which the consensus residues were fixed and the other six positions were randomized [175]. Library screening against D-IQN17 resulted in the 2K-PIE1 peptide which was missing two of the randomized residues (CXXXEWXWLC). The higher binding and inhibitory activity of the new peptides (~4-fold higher than the previous D-peptide) was suggested to be due to the smaller size, which creates a more compact peptide with better packing capability. Therefore, the authors constructed an 8-mer library (CX4WXWLC) and performed a screening against the second-generation trimeric pocket mimic IZN17 protein. Library screening resulted in a PIE7 peptide with more potent inhibitory activity (IC_50_ 620 nM) for HIV-1 strain HXB2 than that of the 2K-PIE1 peptide (IC_50_ 2.2 μM). Dimeric ((PIE7)_2_) and trimeric ((PIE7)_3_) D-peptides were produced by employing bis(NHS ester)PEG crosslinker to conjugate PIE7 via its N- and C-terminal lysines. Both peptides ((PIE7)2 and (PIE7)3) demonstrated higher inhibitory activity with IC_50_ values of 1.9 nM and 250 pM against HXB2, respectively. This dramatic gain in potency was explained with the trimeric structure of gp41 protein. The inhibitory activity of the developed inhibitors was tested against other strains of HIV. While the first-generation D-peptide, D10-p5, showed little or no inhibitory activity against JRFL and a modest activity against BaL, PIE7 inhibited both JRFL and BaL entry, although less potently than HXB2 entry. Low inhibitory activity of the second-generation D-peptides for the JRFL strain made the authors design third-generation inhibitors with possible higher potency to combat diverse primary strains [176]. In 1st- and 2nd-generation PIE inhibitors, four flanking residues were fixed outside the disulfide (Gly–Ala on the N terminus and Ala–Ala on the C terminus). A new phage library was constructed by using a previously optimized PIE7 core sequence (XXCDYPEWQWLCXX) and varying four flanking residues. The library screening against D-IZN17 resulted in a PIE12 peptide with ~40-fold improved inhibitory activity against the JRFL strain compared to the original PIE7 peptide. Similar to the previous PIE7 peptide, dimeric and trimeric forms of PIE12 (IC_50_: 14 and 2.8 nM, respectively) were more potent inhibitors of the JRFL strain as compared to the original monomer PIE12 peptide (IC_50_: 580 nM). 

Fievez et al. also used dimerization and amino acid substitution to enhance the antiviral activity of phage display-selected HIV-1 inhibitors [177]. They constructed a phage display peptide library, a so-called Mimokine library, derived from the N-terminus (aa 1–21) of vCCL2, a broad-spectrum chemokine encoded by human herpesvirus 8. While vCCL2 has high antagonist affinity for CXCR4, its N-terminus has been identified as the shortest peptide sequence with modest CXCR4-binding affinity and antiviral activity. The Mimokine library was constructed by randomizing the amino acids of the vCCL2(1–21) sequence at positions 11 and 12 (Cys11 and Cys12). The library screening against CXCR4-expressing cell lysate resulted in a peptide sequence (Mimokine SR) having SR amino acids instead of cysteine at positions 11 and 12. Dimerization of Mimokine SR was achieved through a cysteine residue added at the C-terminus for disulfide bond formation and increased CXCR4-binding affinity of Mimokine SR which was determined with IC_50_ values of 644 and 41 nM for monomeric and dimeric Mimokine SR, respectively. On the other side, antiviral activity of the dimeric peptide against HIV-1 infection increased 11-fold (IC_50_ = 11 μM) when compared to the monomeric Mimokine SR (IC_50_ = 120 μM). The chirality change in dimeric Mimokine SR by replacing L residues withD amino acids slightly increased both its binding affinity and antiviral activity (IC_50_ = 29 nM and IC_50_ = 7.4 μM, respectively). Aza-β3-amino acid substitution is an alternative approach used to improve the stability of peptides. Aza-β3-amino acids, which have their side chains on a nitrogen atom, exhibit flexible configuration leading to formation of additional hydrogen bonds and enhanced serum stability [178,179]. In fact, the authors replaced serine by aza-β3-octylalanine in monomeric Mimokine SR and observed an improved CXCR4-binding affinity as well as antiviral activity (IC_50_ = 182 nM and IC_50_ = 37 μM, respectively). While additional modification of tryptophan by aza-β3-1-naphthylalanine reduced CXCR4-binding affinity (IC_50_ = 2312 nM), antiviral activity increased significantly (IC_50_ = 9.5 μM). CXCR4-binding affinities and antiviral activities of Mimokine SRs substituted with aza-β3-amino acids were further improved by dimerization. It was achieved via addition of a lysine at position 13 of the peptides.

### 3.3. Conversion of Antibody Fragments into Whole IgG Antibodies

The aforementioned features of recombinant antibody fragments, such as low stability and solubility, as well as short serum half-life, may reduce their activity for in vitro and in vivo neutralization assays. In these cases, conversion of phage display-selected antibody fragments into full IgG antibodies can be employed as a strategy to enhance their biophysical properties [76]. Enhanced neutralizing activity of antibody fragments converted into full-length IgG format is attributed to the longer serum half-life and higher avidity of full-length antibodies [77]. Fusion of the fragment crystallizable region (Fc) to the antibody fragment increases the hydrodynamic radius of the molecule and prevents its rapid renal clearance [108]. Moreover, the uptake of antibodies by the reticuloendothelial system through interaction of the Fc domain with cell receptors protects the antibodies from degradation and increases their serum half-life [108]. Furthermore, dimeric epitope-binding sites of IgG molecules results in higher avidity in comparison to monomeric antibody fragments and leads to higher neutralizing activity [180]. Ohta et al. employed this approach to generate intact human IgG1 antibodies with neutralizing activity against primary human cytomegalovirus (HCMV), a major cause of morbidity and mortality in immunocompromised individuals such as transplant recipients, AIDS patients, and newborn infants [78]. A phage display Fab antibody library was screened against purified HCMV virions and recombinant envelope glycoprotein B in two independent library screenings. While the soluble monovalent formats of selected Fab antibodies did not have any neutralizing activity, intact human IgG antibodies of three clones (H05, H08, and H14) did neutralize HCMV when 5% guinea pig complement was supplemented in assay conditions. Employment of 1.95 μg/mL H05 antibody in clinical isolates sensitive/resistant to the antiviral drug ganciclovir resulted in more than 50% plaque reduction activity.

A similar approach was used to develop human monoclonal antibodies targeting the zoonotic NiV and HeV that can cause severe respiratory illness and febrile encephalitis. M102 Fab was selected by phage display Fab antibody library screening against recombinant glycoprotein of HeV (sG) and exhibited 30% neutralizing activity against both HeV and NiV at 30 μg/mL [79]. Zhu et al. affinity maturated m102 to enhance its cross-reactive neutralizing activity for both NiV and HeV [181]. They constructed an antibody library, based on the original Fab phage display library used to identify m102, by light-chain shuffling combined with heavy-chain VH random mutations introduced by error-prone PCR. Library screening against soluble sG protein of HeV resulted in m102.4, which had better binding affinity than that of m102. Conversion of m102.4 into intact IgG1 resulted in a potent monoclonal antibody with cross-reactive inhibitory activity against NiV and HeV, with IC_50_ values below 0.04 and 0.6 μg/mL, respectively. Moreover, IgG1 m102.4 was more potent than m102.4 Fab in cell fusion inhibition of HeV and NiV. In vivo neutralizing activity of IgG1 m102.4 was studied in a ferret model of acute NiV infection [182]. It was shown that a single infusion of IgG1 m102.4 10 h after NiV challenge fully protected the animals. On day 3, high levels of IgG1 m102.4 were detected in the ferrets and the neutralizing activity was associated with the long serum half-life of antibodies. High antibody concentrations were suggested to sufficiently neutralize the virus and provide a window for the animal immune system to initiate a primary response before antibody-mediated depletion of the virus. Nevertheless, in the pre-group in which the animals were treated with the antibody 24 h before the NiV challenge, only one ferret survived and it had the highest level of m102.4 and the best antibody longevity. This result was also explained by the importance of initially high antibody levels and subsequent longevity in the protection from the disease. The neutralizing activity of IgG1 m102.4 was also studied in a nonhuman primate model of NiV and HeV infection and disease in the African green monkey (AGM) [183]. A high dose of HeV was inoculated by an intratracheal route and mAb was administered by intravenous infusion, mimicking a mucosal challenge and paralleling a systemic treatment scenario. AGMs treated with IgG1 m102.4 at 10 or 24 h after the infection showed either mild or no clinical signs of disease 3 days later, whereas the hematology and clinical chemistry assays were normal. 

Conversion of phage display-selected ScFvs and Fabs into intact IgG antibodies has been employed for development of antivirals against several viruses, including influenza virus, respiratory syncytial virus (RSV), HIV1, coronaviruses, and Venezuelan equine encephalitis virus (VEEV) [80,184,185,186,187,188]. Among them, coronaviruses have gained particular interest due to the severe outbreaks that they caused during the last two decades. For instance, MERS-CoV neutralizing antibodies were selected by screening a naïve phage display Fab library constructed from peripheral blood mononuclear cells (PBMC) of 40 healthy volunteers [189]. The library screening was performed against recombinant RBD of MERS-CoV spike protein and the selected three Fabs (m336, m337, and m338) with the highest binding affinity were converted into IgG1. The RBD-binding affinities of IgG1s were 9.94 × 10^−11^, 8.23 × 10^−10^, and 5.59 × 10^−10^ M for m336, m337, and m338, respectively. While all three antibodies had neutralizing activity for MERS-CoV, m336 demonstrated the most potent inhibitory activity with an IC_50_ of 0.07 μg/mL in Vero cells. The protective efficacy of m336 mAb against MERS-CoV infection was studied in a well-characterized human DPP4 Tg mouse model [190]. The treatment of Tg mice with a single dose of either 1 or 0.1 mg of m336 mAbs 12 h prior to MERS-CoV challenge resulted in 100 and 75% protection against lethality, respectively, compared to the control mice which suffered from profound weight loss and uniform death within days after infection. Therapeutic efficacy of m336 mAbs was also confirmed in a similar experiment in which the single dose of either 1 or 0.1 mg of m336 mAbs were administered 12 h after MERS-CoV challenge. The treatments with 1 and 0.1 mg of m336 mAbs provided 100 and 75% protection, respectively. These data were also supported with lower RNA levels and reduced virus load detected in antibody-treated Tg mice in comparison to control mice. It was suggested that m336 mAb might be a promising therapeutic to provide immediate protection to individuals exposed to MERS-CoV and treat others who have already been exposed.

The latest coronavirus, SARS-CoV-2, has also been a target for antiviral drug discovery studies. Li et al. screened phage display antibody libraries in Fab, ScFv, and VH formats against recombinant RBD of the SARS-CoV-2 spike protein [77]. The highest-affinity binders of each group were converted to IgG1 and VH-Fc fusion formats. IgG1 ab1, selected from the Fab phage library, demonstrated the highest neutralizing activity for SARS-CoV-2 pseudovirus. IC_50_ of IgG1 ab1 was 200 ng/mL and antibody-dependent cellular cytotoxicity (ADCC) was observed at moderate levels (10 to 15% cell killing). The efficacy of IgG1 ab1 in vivo was evaluated using one hamster model and two mouse models of SARS-CoV-2 infection. One of the mouse models was ACE2-adapted SARS-CoV-2 with two mutations, Q498T/P499Y, at the interface of RBD and ACE2. While the virus neutralization was dose-dependent, the complete neutralization was observed at the highest dose of 36 mg/kg. These results suggest that the double mutation Q498T/P499Y on RBD does not affect IgG1 ab1 binding. The second model was hACE2-expressing transgenic mouse and administration of IgG1 ab1 to the mice prior to wild-type SARS-CoV-2 challenge inhibited the virus propagation in four of the five mice. For both models, a 100-fold reduction in virus in the lungs was achieved with similar doses of antibody (10 to 15 mg/kg). In a hamster model of SARS-CoV-2 infection, the intraperitoneal administration of 10 mg/kg IgG1 ab1 prior to an intranasal virus challenge led to a 10,000-fold reduction in virus titer in the lungs of four out of five hamsters at day 5 post infection. Of note, the 6 h post-challenge intraperitoneal administration of IgG1 ab1 was three times more efficient at decreasing infectious virus titer as compared to prophylactic administration. These results suggest the potential use of IgG1 ab1 both for prophylaxis and therapy of SARSCoV-2 infection. In the report of Noy-Porat et al., a phage display ScFv library was constructed from the PBMCs of two patients at the acute phase of COVID-19 [191]. The independent library screenings against recombinant mFc-S1 and huFc-RBD proteins resulted in eight different ScFvs (MD17, MD29, MD45, MD47, MD62, MD63, MD65, and MD67) carrying unique sequences. ScFv-Fc were classified into four groups that each recognized distinct epitopes: I (MD17, MD29, and MD63); II (MD45, MD65, and MD67); III (MD62), and IV (MD47). In neutralization assays using VeroE6 cells infected with SARS-CoV-2, pre-incubation of the virus with MD65 antibody resulted in the highest neutralizing activity. The neutralization dose needed to inhibit 50% of the plaques (NT_50_) was of 0.22 μg/mL. SARS-CoV-2-specific antibodies were also identified by Li et al. in a screening of the V_H_ phage display library against recombinant RBD protein [82]. The human V_H_ phage display library was constructed by grafting heavy-chain CDR1, 2, and 3 genes derived from 12 healthy donors’ PBMCs and splenocytes into a stable scaffold consisting of engineered germline V_H_3-23. V_H_ ab8, exhibiting one of the highest RBD-binding affinities as well as high stability in Dulbecco’s phosphate-buffered saline, was converted to a bivalent antibody domain by fusion to the human IgG1 Fc (V_H_-Fc ab8). The SARS-CoV-2 RBD-binding affinity of V_H_-Fc ab8 (K_D_, 0.54 nM) was higher than that of V_H_ ab8 (19 nM) due to the increased avidity. The authors also showed a similar binding affinity of V_H_-Fc ab8 to mutant RBDs (F342L, N354D, N354D/D364Y, V367F, R408I, W436R, K458R, G476S, and V483A) in ELISA. Inhibitory activity of V_H_-Fc ab8 was determined in pseudovirus assays in which it outperformed ACE2-Fc with IC_50_ values 0.03 and 0.40 mg/mL, respectively. Prophylactic efficacy of V_H_-Fc ab8 was investigated in a mouse model, in which wild-type BALB/c mice were challenged with SARS-CoV-2 carrying two mutations Q498T/P499Y in the RBD. Administration of V_H_-Fc ab8 prior to SARS-CoV-2 challenge effectively inhibited the virus in a dose-dependent manner and reduced viral RNA in the lungs of the mouse. Prophylactic and therapeutic efficacy of V_H_-Fc ab8 was also studied in a hamster model. Both prophylactic and therapeutic administrations reduced viral RNA in the lung and alleviated pneumonia. V_H_-Fc ab8 had pharmacokinetics comparable to that of a full-size IgG1 demonstrated by its persistence at significant levels for 4 days post administration.

### 3.4. Modification of Antiviral Peptides/Antibodies for Intracellular Delivery

Introduction of large hydrophilic molecules such as peptides and proteins into the cytosol is a challenging task hindered by the cell membrane, which represents a non-permissive barrier [192]. However, intracellular delivery of antiviral molecules targeting cytoplasmic proteins is highly desirable to observe their inhibitory effects on infected cells [193]. In this respect, cell-penetrating peptides (CPP), also known as protein transduction domains (PTDs), have been widely used to transport antivirals across the cell membrane without disturbing the membrane structure [194]. Penetratin, derived from the third helix of the Drosophila Antennapedia homeodomain (RQIKIWFQNRRMKWKK), has been one of those CPPs used to deliver antivirals into the cells [195]. Yang et al. produced penetratin fusions of peptide sequences (Ant-VMI7 and Ant-VMI9) targeting recombinant virion infectivity factor (Vif) protein, which is essential for HIV-1 replication [196]. As the cytoplasm is the main location of Vif in infected cells, the penetratin fusion enhanced the inhibitory activity of the peptides by facilitating their efficient delivery into cells. Administrations of Ant-VMI7 and Ant-VMI9 fusion peptides at 50 μM concentration were able to inhibit HIV-1 replication in H9 cells. The inhibition mechanism was suggested to be due to the interference of the selected peptides with Vif multimerization, which is required for Vif function. In another report, fusion of penetratin was employed to produce cell-penetrable nanobodies (so-called transbodies) to inhibit HCV replication in human hepatic (Huh7) cells [197]. Selected nanobodies targeted the nonstructural NS4B protein, which plays pivotal roles in HCV life cycle and pathogenesis. The treatment of HCV RNA-transfected Huh7 cells with transbodies significantly reduced the amount of HCV mRNA and restored the virally suppressed host innate immune response as assessed by upregulation of the expressions of innate cytokine genes (IRF3, IFN-β, and IL-28b). 

The Tat peptide is another promising CPP widely studied for systemic administration of therapeutics [198]. It is derived from the HIV-1 transactivator of transcription (Tat) protein which can efficiently enter the cells and thus mediate the transport of biological macromolecules through the cell membrane to achieve their physiological function [199]. Zhuang et al. studied the effect of the Tat protein on the antiviral activity of FabRev1, selected by screening of the phage display Fab library against recombinant HIV-1 Rev protein [200]. Although FabRev1 and its Tat fusion product, FabRev1–Tat, had similar HIV1 Rev-binding affinities (*K_d_*; 6.8 × 10^−10^ and 5.0 × 10^−10^ M, respectively), antiviral activity was dependent on the fusion of Tat peptide and observed only with FabRev1–Tat. The authors showed that the fusion of the Tat peptide enabled the entry of FabRev1 to PBMC where it could interfere with Rev oligomerization and inhibit the replication of HIV1. FabRev1–Tat could inhibit the infection of three different CCR5 isolates with IC_50_ values determined in the range of 0.09 to 0.44 μg/mL.

Short polyarginine peptides are another group of CPPs which have been used for cellular delivery of antivirals targeting nonstructural viral proteins such as NS5A of HCV and NS1 of DENV [201,202]. The enhanced ability of arginine-rich peptides to cross the cell membrane is attributed to the presence of the guanidine head group in the side chain of arginine [203]. In fact, the interaction of polyarginine peptides with the cell membrane occurs through the hydrogen bonds formed between the positively charged guanidine group and the negatively charged phosphates and sulfates on the cell surface [204]. Phanthong et al. used nanoarginine (R9) peptide to enhance the cell penetration ability of HuscFv27 antibody, selected against the structural VP4 protein of enterovirus 71 (EV71), one of the viruses causing hand, foot, and mouth disease (HFMD) [205]. The externalized VP4 protein forms a membrane pore that enables the exit of viral RNA into the cytoplasm to initiate viral translation and replication. The authors showed that HuscFv27 antibody with R9 peptide fusion (R9–HuscFv27) could enter any infected/uninfected rhabdomyosarcoma (RD) cells due to enhanced cell permeability. However, HuscFv27 antibodies could enter only EV71-infected RD cells, which was explained with the increased plasma membrane permeability of infected cells. Moreover, both antibodies inhibited VP4 pore-forming activity, while the inhibitory activity of HuscFv27 was the highest. It was suggested that the ability of HuscFv27 antibody to enter only infected cells led to higher inhibitory activity due to an accumulation of antibodies in these cells. Conversely, R9–HuscFv27 antibodies could enter and exit any cells whether they contained the target protein or not, which led to their lower inhibitory activity. 

## 4. Perspective

Phage display has been one of the most powerful drug discovery technologies to lead to the development of FDA-approved peptide and antibody drugs for various diseases (e.g., hereditary angioedema, immune thrombocytopenic purpura, rheumatoid arthritis, and uveitis) [33,45,206] The success of phage display-derived drug molecules as well as the growing interest in peptides/antibodies in the biopharmaceutical market make phage display technology a popular approach for antiviral discovery. In fact, since the beginning of the latest coronavirus outbreak, several research groups have screened phage libraries to develop neutralizing recombinant antibodies against SARS-CoV-2 [83,191,207,208,209,210,211,212].

Despite the success of phage display in drug discovery, peptides and antibody fragments still suffer from several challenges, as well as misconceptions, limiting their entry into the clinic. While poor proteolytic stability and low oral bioavailability result in poor pharmacokinetics, hindering the use of peptides as therapeutics, antibody fragments cannot compete yet with full-length antibodies in terms of serum half-life and potency [137,213]. However, as summarized in this review, there are various approaches available to enhance the pharmacokinetics of peptides and antibody fragments. Chemical modification of peptides for improved proteolytic stability and conversion of antibody fragments into full-length antibodies for longer serum half-life are only a few of them. Indeed, the delay in the realization of peptides in the drug market is suggested to be due to misconceptions, such as high production cost, poor pharmacodynamics, and lack of oral bioavailability [214]. Although the high cost of large-scale peptide production is evident, Otvos and Wade emphasized that the cost of active pharmaceutical ingredients makes up a small percentage of total drug development cost (<3%) and it is fairly compensated by the relatively high clinical success rate of peptides [214]. In addition, different delivery options (e.g., nasal and oral) are available for peptide drugs upon enhancement of their pharmacokinetics by chemical/physical modifications; however, it is necessary to increase the awareness of drug developers about delivery options to eliminate this misconception [214,215,216]. On the other side, there are only a few antibody fragments in the market despite their several benefits over full-length antibodies (e.g., new administration routes, ability to target epitopes inaccessible to traditional antibodies, and cost-efficient production) [217]. Experts suggest that the approval of caplacizumab, the first nanobody approved by the FDA in 2019, has already increased the interest in domain antibodies and that their use in applications in which they can outperform traditional antibodies will make them reach their full potential and expand faster in the drug market [137]. 

Lastly, it is important to note the specific state of antiviral biologics in the drug market. Currently, there are few approved antiviral peptide/antibody products (e.g., enfuvirtide, boceprevir, telaprevir, palivizumab, and ibalizumab) [35,107]. Besides the aforementioned challenges encountered by peptides and antibodies, antiviral drug development encounters additional difficulties, slowing down their process, such as occurrence of neutralization escape mutations and development of effective vaccines offering life-long immunity [218,219]. Short duration of viral illnesses is another obstacle making antiviral drugs less attractive commercial products for the market [220]. However, certain conditions such as low vaccine coverage in society and lack of vaccines for some viruses still necessitate the development of effective antivirals. The growing market of peptide/antibody drugs also suggests an increase in delivery of new biologic antivirals in the near future.

## Figures and Tables

**Table 1:** A list of selected phage display-derived antivirals.

**Peptides**					
**Peptide Sequence**	**Virus**	**Targeted Antigen**	**Binding Affinity**	**Inhibitory Activity (IC_50_)**	**Ref**
SENRKVPFYSHS	JEV	Domain III of envelope protein	6.06 × 10^−6^ M (*K_d_*)	~1 μM	[55]
CNDFRSKTC	H9N2	Intact virus	NA	48 μM	[68]
ACFPWGNQWCGGK	HCV	RNA-dependent RNA polymerase, NS5B	34 μM (*K_d_*)	8.82 μM	[69]
CDVIALLACHLNT	WNV	Envelope protein, E	6 μM (*K_d_*)	2.60 ± 0.01 μM	[70]
KHMHWHPPALNT	HBV	PreS1 protein	7.21 × 10^4^ ± 4.15 × 10^4^ M^−1^ (*K_a_*)	NA	[71]
ITFEDLLDYYGP	HIV-1	Capsid domain of Gag polyprotein	15.0 ± 7.2 μM (*K_d_*)	NA	[72]
RAVWRHSVATPSHSV	H1N1	Neu5Ac	0.41 μM (*K_d_*)	6.5 μM	[73]
**Antibody Fragments**					
**Antibody Fragment**	**Virus**	**Targeted Antigen**	**Binding Affinity**	**Inhibitory Activity (IC_50_)**	**Ref**
Fab	HEV	Putative capsid protein, ORF2	1.7 nM (*K_d_*)	NA	[74]
Fab	Marburg virus	VP35 protein	4.9 ± 1 nM (*K_d_*)	NA	[75]
Fab	EBOV	Envelope glycoprotein (GP)	NA	1 μM	[76]
Fab	SARS-CoV-2	RBD	1.5 nM (*K_d_*)	NA	[77]
Fab	HCMV	Glycoprotein B	9.3 nM (*K_d_*)	NA	[78]
Fab	HeV	Envelope glycoprotein, G	28 nM (*K_d_*)	4.2 μg/mL	[79]
Fab	HIV-1	Envelope glycoprotein (gp140)	1.4 nM (*K_d_*)	8 μg/mL	[80]
VH	SARS-CoV-2	S1 subunit of spike protein	3.70 ± 0.09 nM (*K_d_*)	2.6 μg/mL	[81]
VH	SARS-CoV-2	RBD	19 nM (*K_d_*)	0.65 μg/mL	[82]
VHH	SARS-CoV-2	RBD	21.6 nM (*K_d_*)	0.55 μg/mL	[83]

Abbreviations: JEV, Japanese encephalitis virus; H9N2, avian influenza virus subtype; WNV, West Nile virus; HCV, hepatitis C virus; HBV, hepatitis B virus; HIV-1, human immunodeficiency virus 1; H1N1, avian influenza virus subtype; HEV, hepatitis E virus; EBOV, Ebola virus; SARS-CoV-2, Severe acute respiratory syndrome coronavirus-2; HCMV, human cytomegalovirus; HeV, Hendra virus; RBD, receptor-binding domain; Fab, antibody-binding fragment; VH, variable domain of heavy chain; VHH, variable heavy chain of HCAb; NA, not available.
